# Neutrophil extracellular traps and von Willebrand factor are allies that negatively influence COVID‐19 outcomes

**DOI:** 10.1002/ctm2.268

**Published:** 2021-01-01

**Authors:** María P. Fernández‐Pérez, Sonia Águila, Laura Reguilón‐Gallego, Ascensión M. de los Reyes‐García, Antonia Miñano, Carlos Bravo‐Pérez, María E. de la Morena, Javier Corral, Nuria García‐Barberá, José M. Gómez‐Verdú, Enrique Bernal, María T. Herranz, Vicente Vicente, Constantino Martínez, Rocío González‐Conejero, María L. Lozano

**Affiliations:** ^1^ Department of Hematology and Medical Oncology Hospital General Universitario Morales Meseguer, Centro Regional de Hemodonación, Universidad de Murcia, IMIB Murcia Spain; ^2^ CIBERER U765 Murcia Spain; ^3^ Department of Internal Medicine Hospital General Universitario Morales Meseguer Murcia Spain; ^4^ Department of Infectious Diseases, Hospital General Universitario Reina Sofía Universidad de Murcia, IMIB Murcia Spain

Dear Editor,

Complex interactions between various processes have arisen as key elements underlying the pathophysiology of coronavirus disease 2019 (COVID‐19). First, immunothrombosis and neutrophil extracellular trap (NET) release contribute to inflammation‐associated lung damage and thrombosis, and second, endothelial cell dysfunction participates in the disease‐associated coagulopathy.^1^, ^2^ In addition, cytokine release syndrome (CRS) dictates adverse clinical outcomes, with interleukin (IL)‐6 playing a central role.^3^ However, the sequence of events leading to a vicious cycle of synergistic pathways causing unchecked immune activation and thrombus formation have to date not been clearly defined.

Here, we investigated the role of NETs, endotheliopathy, hemostatic imbalance, and interleukin‐6 (IL‐6) release in the severity of the disease, as well as their synergy in patient outcomes. For this purpose, we recruited 142 hospitalized COVID‐19 patients from March to May 2020. Infection was confirmed by qRT‐PCR testing. Healthy controls (HC, N = 50) were also included. Clinical and demographical variables are reported in Table S1, and biochemical analytical data and cell counts in Table S2.

To address whether NETs and von Willebrand factor (VWF)/a disintegrin‐like and metalloprotease with thrombospondin type 1 motif 13 (ADAMTS13) axis are associated and contribute to the prognosis of COVID‐19 patients, we analyzed plasma cell‐free DNA (cfDNA), citrullinated histone H3‐DNA (citH3‐DNA) complexes (surrogated NET marker), von Willebrand factor antigen (VWF:Ag), von Willebrand factor collagen binding (VWF:CB), ADAMTS13 activity, IL‐6 levels, clinical scores, and outcomes. Analysis of NETosis and endotheliopathy markers in the first available sample confirmed a coagulation imbalance in COVID‐19 patients versus HC (Figure S1A‐F). In addition, NETs and VWF:Ag correlated with neutrophil count, neutrophil/lymphocyte ratio, and with acute‐phase reactants and coagulation parameters (Table S3). Importantly, NETs and VWF:Ag/ADAMTS13 ratio measured in the first available sample seemed to account for severity, given their association with clinical scores (Figure 1A‐F and Figure S2A‐F), hypercoagulable state (Figure1G‐I and Figure S2G‐I), acute respiratory distress syndrome (Figure1J‐L and Figure S2J‐L), and intensive care unit (ICU) admission (Figure1M‐O and Figure S2M‐O). Importantly, VWF:Ag levels were strongly correlated with cfDNA and with citH3‐DNA in the first available sample (Figure S3A). Considering cfDNA levels as a surrogate marker of cell damage, we analyzed the correlation between NETosis and VWF. At maximum cfDNA values, the correlation between NET markers and VWF:Ag was maximal (Figure S3B), but nonexistent at trough cfDNA levels (Figure S3C). The dynamics of cfDNA, citH3‐DNA, and VWF:Ag showed that VWF rose together with NET markers, but it kept increasing in spite of the decrease of the latter variables (Figure 2A). Evolution of Spearman's rho correlation coefficients at three time points is shown in Figure 2B. Altogether, these data support causality among these parameters and disease aggravation. Reinforcing this, we found that in a small group of eight patients both NET and VWF levels continued to increase once patients entered the ICU (Figure 2C‐E). Remarkably, patients with lower ADAMTS13 activity (<63%) had lower survival (Figure 2F). Moreover, higher VWF:Ag/ADAMTS13 activity ratio in the first available sample was associated with mortality (Figure 2G) as it has been described for ischemic stroke.^4^ADAMTS13 is crucial in the maintenance of vascular homeostasis upon infections and its activity has been described to be lower in sepsis than in noninfectious shock.^5^ It was striking that up to 18.3% of patients had a severe decrease (<60%) in ADAMTS13 activity (Figure S1E). In sepsis, ADAMTS13 has been found highly citrullinated, a posttranscriptional modification implicated in NET formation, due to the action of peptidylarginine deiminase type IV (PAD4).^6^ Interestingly, we found that severe decreased ADAMTS13 activity was inversely correlated with citH3‐DNA levels in plasma (Figure S4). Thus, we hypothesize that low ADAMTS13 activity observed in COVID‐19 patients might be due in part to high levels of PAD4, or by the action of specific autoantibodies.^7^ Overall, these results are in agreement with previous reports associating NETs and VWF with severity in COVID‐19.^1^, ^2^, ^8^ Additionally, our study showed that VWF/ADAMTS13 imbalance contributed to adverse outcomes, influencing patient's survival.

**FIGURE 1:**
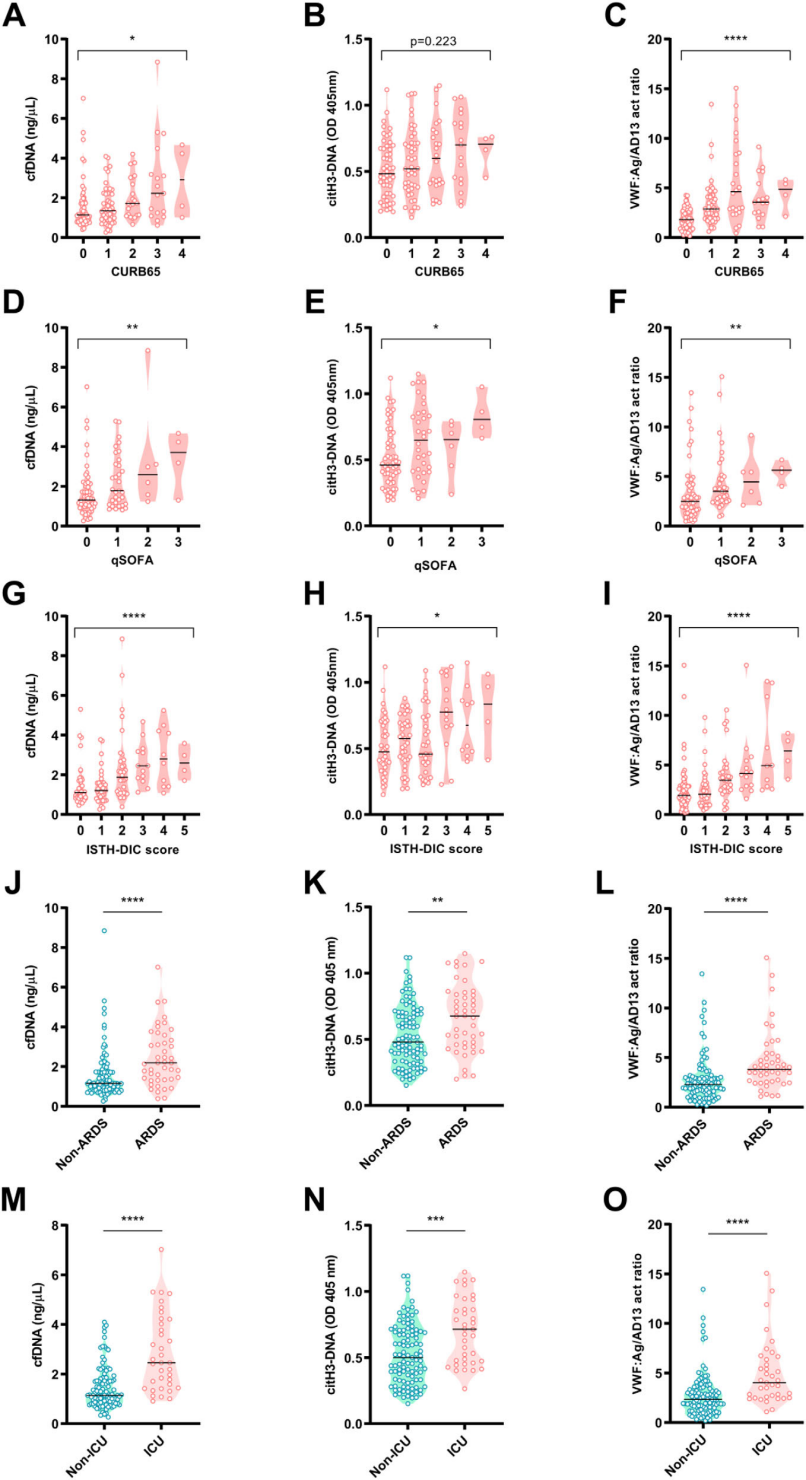
Severity and coagulopathy scores, ARDS development, and need for ICU admission were associated with NET markers and VWF/ADAMTS13 axis imbalance. The following markers were measured in the first available plasma sample from COVID‐19 patients and analyzed according to CURB‐65 score (A‐C): (A) cfDNA, (B) citH3‐DNA, and (C) VWF:Ag/ADAMTS13 activity (AD13 act) ratio; qSOFA score (D‐F): (D) cfDNA, (E) citH3‐DNA, and (F) VWF:Ag/ADAMTS13 activity ratio; and maximum ISTH‐DIC score reached during follow‐up (G‐I): (G) cfDNA, (H) citH3‐DNA, and (I) VWF:Ag/ADAMTS13 activity ratio. Kruskal‐Wallis test was used for statistical analysis. The same markers were compared in plasma samples from ARDS versus non‐ARDS COVID‐19 patients (J to L): (J) cfDNA, (K) citH3‐DNA, and (L)VWF:Ag/ADAMTS13 activity ratio; and from ICU versus non‐ICU patients. (M‐O): (M) cfDNA, (N) citH3‐DNA, and (O) VWF:Ag/ADAMTS13 activity ratio. Mann‐Whitney *U*‐test was used for the statistical analysis. *P < .05, **P < .01, ***P < .001, ****P < .0001

**FIGURE 2:**
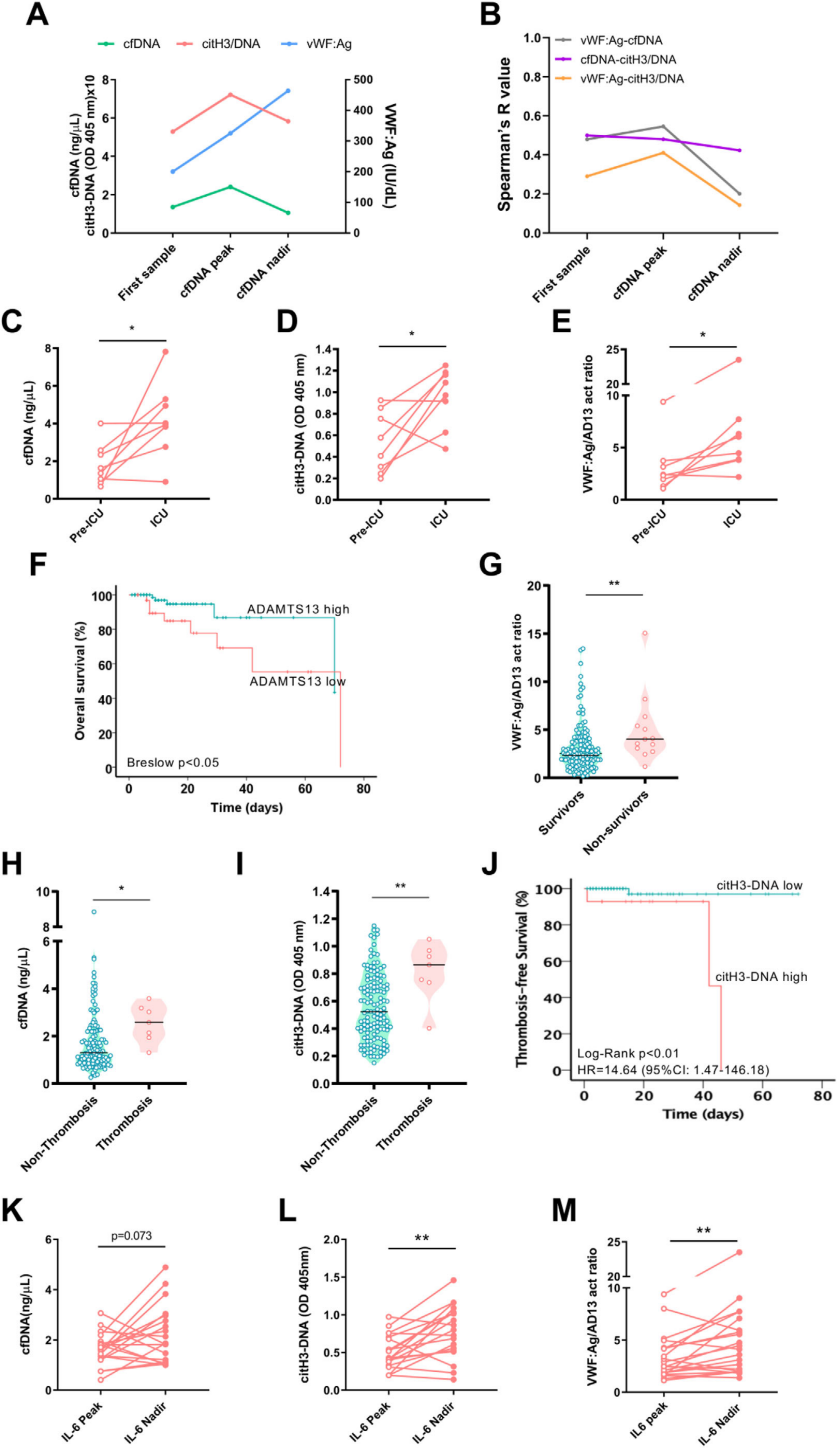
NET and VWF/ADAMTS13 axis markers increase with COVID‐19 severity following IL‐6 peak, and predict adverse outcomes. (A) Dynamics of plasma cfDNA, citH3‐DNA, and VWF:Ag median levels at three time points (first available sample, cfDNA peak, and cfDNA nadir) in COVID‐19 patients indicated that NET markers and VWF:Ag elevation occurred simultaneously, while the decrease of VWF:Ag occurred afterwards. (B) Correlation among cfDNA, citH3‐DNA, and VWF:Ag at these three moments. Spearman's rho correlation test was performed for statistical analysis. Plasma (C) cfDNA, (D) citH3‐DNA complexes, and (E) VWF:Ag/ADAMTS13 activity (AD13 act) levels changes from general hospitalization to ICU admission (N = 8). Wilcoxon test was used to analyze the statistical differences between matched paired samples. (F) Kaplan‐Meier survival curves with Breslow test for high versus low ADAMTS13 activity (cutoff point = 63.25%) in the first available sample of COVID‐19 patients showing reduced survival in the low ADAMTS13 activity group. ROC curve was used to determine the most appropriate cut‐off value of ADAMTS13 activity. (G) VWF:Ag/ADAMTS13 activity ratio in nonsurvivor versus survivor COVID‐19 patients. Mann‐Whitney *U*‐test was used for the statistical analysis. (H) cfDNA levels and (I) citH3‐DNA complexes in the first available plasma sample were associated with thrombotic events (N = 7). Mann‐Whitney *U*‐test was used for statistical analyses. (J) Kaplan‐Meier curve with log‐rank (Mantel‐Cox) test comparing time to thrombosis between patients with high and low citH3‐DNA levels taking into account plasma samples collected before the thrombotic event (four available samples of the seven thrombosis patients). Patients with citH3‐DNA complexes higher than OD cutoff point 0.903 had an increased thrombotic risk (HR = 14.64; 95%CI: 1.47‐146.18). ROC curve was used to determine the most appropriate cut‐off value of citH3‐DNA complexes. The prognostic effect of citH3‐DNA complexes was assessed using Cox proportional hazard regression model. The following markers were measured in plasma from COVID‐19 patients in the IL‐6 peak and in the IL‐6 nadir (N = 19): (K) cfDNA, (L) citH3‐DNA complexes, and (M) VWF:Ag/ADAMTS13 activity ratio. Wilcoxon test was used to analyze the statistical differences between matched paired samples. *P < .05, **P < .01, ***P < .001, ****P < .0001

Since both the magnitude of the thrombotic complications and their impact on mortality are distinctive signs of this disease, we investigated the association between thrombosis and NET markers. Our findings in seven patients with proven thrombosis confirmed such an association (Figure 2H‐I). The hypercoagulable state related to citH3‐DNA at early stages of hospitalization (Figure 1H) evolved toward increased thrombotic risk, suggesting that high NET levels might predict future thrombotic complications (Figure 2J).

Another pathognomonic sign of COVID‐19 is CRS, which seems to be mediated by an inflammation circuit due to IL‐6 trans‐signaling causing endotheliopathy.^9^ Thus, we evaluated if there was a temporal association between NET and VWF/ADAMTS13axis dynamics, and IL‐6 levels. For this, we analyzed those parameters in the maximal and nadir values of IL‐6 in 19 patients. Interestingly, our data showed that IL‐6 levels increase occurred earlier in time than that of NETs and VWF (Figure 2K‐M and Figure S5A‐C), an observation that may have an impact on the therapeutic management of these patients. Taken together, these results suggest that cytokine storm is the triggering event in COVID‐19, which is followed by NETosis and endothelial cell activation together with the hemostatic imbalance that correlate with clinical outcomes. Different studies have provided evidences of the connection between neutrophils/NETs, CRS, and VWF/ADAMTS13: (i) α‐defensins, released from neutrophils, bind to A2 domain of VWF inhibiting its proteolytic cleavage by ADAMTS13; (ii) VWF directly binds to leukocytes and NETs; (iii) IL‐6 inhibits VWF cleavage by ADAMTS13 in vitro; (iv) CRS is exacerbated in inflammatory diseases when abnormal NET levels are generated; and finally (v) histones from NETs induce VWF release from endothelial cells in vitro and in vivo.^10^

In conclusion, our results support that the increase of IL‐6 due to the CRS synchronizes a feedback mechanism between NETosis and VWF/ADAMTS13 axis, reflected by the correlation dynamics between plasma levels of NETs and VWF that, in turn, may perpetuate endothelium damage, amplifying immunothrombosis and worsening the course of the disease (Figure 3). Additional studies are warranted to confirm the potential use of VWF and ADAMTS13 activity as predictive markers of clinical outcomes. The present data may support that the timing for new therapeutic opportunities targeting CRS, NET formation/removal, and VWF/ADAMTS13 axis imbalance may have an impact on patient's prognosis.

**FIGURE 3:**
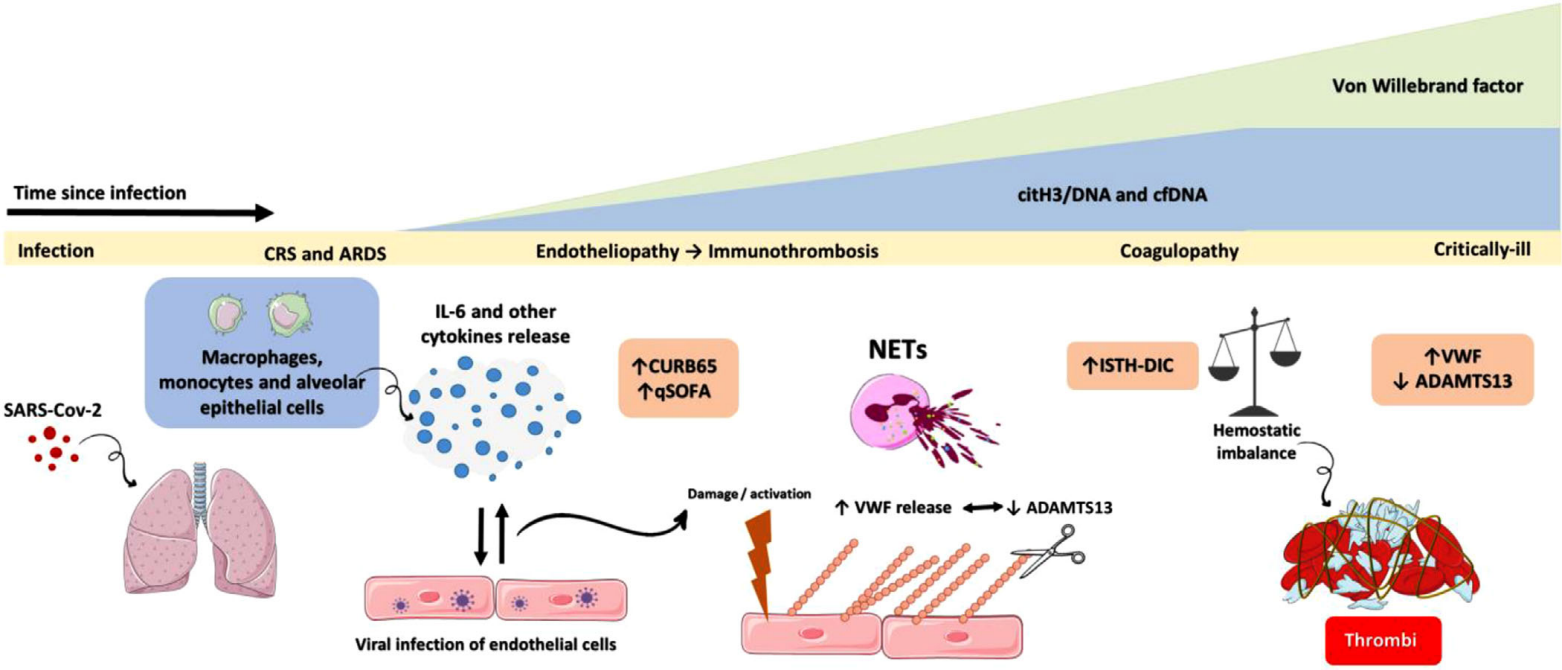
Proposed time‐course phenomena favoring COVID‐19 progression related to the synergistic effect between NET formation and VWF/ADAMTS13 imbalance

## CONFLICT OF INTEREST

The authors declare that there is no conflict of interest.

## AUTHOR CONTRIBUTIONS

MPF‐P, SA, AMDLR‐G, and NG‐B performed research; MPF‐P, SA, CM, and RG‐C performed data analysis; LRG, CB‐P, EB, JMG‐V, MTH, and MLL supervised and collected the clinical data; AM, MEM‐B, and JC collected the samples; MPF‐P, SA, CM, and RG‐C wrote the manuscript; VV and MLL supervised the study.

## ETHICS APPROVAL

All participants or their legal authorized representatives gave written informed consent for study enrollment in accordance with the Declaration of Helsinki. Study was approved by local ethic committee, Morales Meseguer Hospital C.P. ‐ C.I. EST: 24/20.

## CONSENT FOR PUBLICATIONS

All the authors consent for publication.

## Supporting information

Supporting Information

## Data Availability

All the data obtained and/or analyzed during the current study are available from the corresponding authors on reasonable request.
