# Supplementary material for: Neutrophil extracellular traps and von Willebrand factor are allies that negatively influence COVID‐19 outcomes
Source: Clin Transl Med. 2021 Jan 1;11(1):e268. doi: 10.1002/ctm2.268 (PMC7775985; doi:10.1002/ctm2.268)
Supplement: Supplementary file 1 — Supporting Information [file CTM2-11-e268-s001.docx]

**Neutrophil extracellular traps and von Willebrand factor are allies that negatively influence COVID-19 outcomes**

Fernández-Pérez et al

**Supporting Information:**

Supporting Patients and Methods

Supporting Tables

Supporting Figures

SUPPORTING PATIENTS AND METHODS

COVID-19 patients and controls

The study was performed in a cohort of 142 consecutive patients with COVID-19 requiring admission in Morales Meseguer and Reina Sofia University Hospitals (Murcia, Spain). SARS-CoV-2 infection was confirmed by quantitative real-time reverse-transcriptase polymerase chain reaction (qRT-PCR) testing. We collected first and consecutive available samples of each patient as citrate-anticoagulated whole blood from March to May 2020. The median time between hospital admission and first available sample was 2 (IQR 1-7) days. Platelet-poor plasma (PPP) was isolated by centrifugation of whole blood (1500g x 10 min at RT). Aliquots were stored at -80ºC to allow batch analysis. The study was approved by the Ethics Committee of Morales Meseguer University Hospital.

All participants or their Legal Authorized Representatives gave written informed consent for study enrollment in accordance with the Declaration of Helsinki. Also, healthy blood donors were included. All participants were older than 18.

Data collection

Clinical and routine laboratory data from COVID-19 patients were retrospectively collected from medical records with a median follow-up of 108 (IQR 102-112) days. Clinical information included patient demographics, medical comorbidities, prior history of thrombosis, chronic anticoagulation, hospital length of stay, CURB-65 and qSOFA scores, development of ARDS, thrombotic/hemorrhagic events, ICU admission, and death. Venous and arterial thrombotic episodes were confirmed by radiologic methods [Doppler-Ultrasonography (US) and Computed Tomography (CT)].

Routine laboratory parameters were recorded from first available sample, including values of different plasma markers of cell lysis [lactate dehydrogenase (LDH)] and systemic inflammation [C reactive protein (CRP), IL-6, procalcitonin (PCT)]; complete blood cell counts and basic coagulation tests [prothrombin time (PT), activated partial thromboplastin time (aPTT), fibrinogen, and D-dimer]. COVID-19 associated coagulopathy was graded according to disseminated intravascular coagulation (DIC) score of the International Society on Thrombosis and Haemostasis (ISTH)18.

Measurement of NETosis markers

Cell free DNA (cfDNA) was quantified using 2µM Sytox® Green reagent (ThermoFisher, Madrid, Spain). DNA from salmon sperm (Sigma-Aldrich, Madrid, Spain) was used to generate the standard curve for Sytox measurements. Fluorescence was read at 488 nm in a microplate reader (Biotek® Sinergy TM Ht, Winooski, VT).

Citrullinated histone 3 (citH3)-DNA complexes were measured in the same samples via sandwich ELISA, as previously described 19. Briefly, rabbit anti-citH3 (Abcam, Madrid, Spain) was used as capture antibody and, after blocking, plasma samples were added to wells and incubated for 2h at RT. Wells were washed and incubated with peroxidase-conjugated anti-DNA antibody (Cell Death Detection ELISA Kit, Roche Applied Science, Indianapolis, IN) for 2h at RT. Optical density (OD) at 405 nm was used as a measure of citH3-DNA complexes abundance two hours after the addition of ABTS (ThermoFisher, Madrid, Spain) as peroxidase substrate.

VWF:Ag ELISA

VWF:Ag was measured by ELISA using rabbit anti-human VWF antibody (Dako, Agilent Technologies, Santa Clara, CA). Briefly, Maxisorp plates (NuncThermoFisher, Madrid, Spain) were coated with anti-VWF antibody diluted 1/1000 in carbonate buffer. Then, serial dilutions of plasma and reference plasma were incubated for 2h following the blocking step with 3% BSA in PBS-Tween 0.1% for 1h. After washing with 0.1% PBS-T, the rabbit anti-human VWF/HRP antibody (Dako, Agilent Technologies, Santa Clara, CA) diluted 1/1000 was added and OD at 492 nm was measured after adding OPD (ThermoFisher, Madrid, Spain) as peroxidase substrate.

VWF collagen binding (VWF:CB)

The collagen-binding activity of VWF was measured by ELISA. Briefly, MaxiSorp plates (Nunc Thermo Fisher, Madrid, Spain) were coated with 5 µg/mL collagen human type III derived from placenta (Sigma-Aldrich, Madrid, Spain) in carbonate buffer. Wells were then blocked with 2.5% BSA in PBS. After washing, serial dilutions of plasma samples and reference plasma were incubated for 2h at room temperature. Plate was washed and incubated with HRP-conjugated rabbit anti–human VWF/HRP antibody (Dako, Agilent Technologies, Santa Clara, CA) diluted 1/1000 in PBS-T. OD at 492 nm was measured after adding OPD as peroxidase substrate.

Quantification of ADAMTS13 activity

FRETS VWF-73 (Anaspec, Fremont, CA) was used to quantify the ADAMTS13 activity from controls and COVID-19 patients. Plasma samples (4 µl) were diluted in 100 µl of reaction buffer (5 mMBis-Tris, 25 mM CaCl2, and 0.005% Tween-20 at pH 6.0) in a white plate. Reference plasma was also diluted in the same buffer for the standard curve. Substrate solution (100 µl; 2µM final concentration) were added to each well. The fluorescence (λex=340nm λem=450nm) was registered every 5 min during 1h at 30°C, and the slope was calculated by linear regression.

Statistics

Descriptive analysis of qualitative and quantitative variables included frequency distributions with percentages, mean ± standard deviation (SD), median with interquartile range (IQR), and 95% Confidence Intervals (95% CI), when appropriate. Kolmogorov-Smirnov and Shapiro-Wilk tests were used for testing normality. Spearman’s correlation coefficient was used to measure the strength of association between two variables and the direction of the relationship. Mann-Whitney U-test was used for median comparison between two independent groups. Kruskal-Wallis test by ranks was used for median comparison between more than two independent groups. Wilcoxon signed-rank test was used for related samples comparison. Receiver Operating Characteristic (ROC) curves were used to determine the most appropriated cutoff value of continuous covariates. Kaplan-Meier method with Log-rank (Mantel-Cox) Test was used to compare differences in time to thrombosis between patients with high and low citH3-DNA. The predictive value for thrombosis was assessed using Cox proportional hazard regression model. Kaplan-Meier method with Breslow correction was applied for survival analysis of patients with low vs. high ADAMTS13 activity.

Statistical analysis was performed with the use of Excel (Microsoft, Redmond, WA), GraphPad Prism 8.0 (GraphPad Software, La Jolla, CA), and IBM SPSS Statistics 25 (IBM Corp., Armonk, NY).

SUPPORTING TABLES

|  | | COVID-19  N=142 | HEALTHY CONTROLS  N=50 |
| --- | --- | --- | --- |
| **Demographics** | | | |
| Sex, females N (%) | | 64 (45.1) | 22 (44) |
| Age, median (IQR) | | 60 (50-74) | 51 (48-57) |
| Blood group, O-group N/valid N (%) | | 46/101 (45.5) | 20/50 (40) |
| **Hospitalization data** | | | |
| Hospital stay, median days (IQR) | | 10 (5-19) | - |
| ICU, N (%) | | 37 (26.1) | - |
| ICU stay, median days (IQR) | | 14 (8-26) | - |
| **Comorbidities, N/valid N (%)** | | | |
| Hypertension | | 60/129 (46.5) | - |
| Diabetes mellitus | | 24/129 (18.6) | - |
| Dyslipidemia | | 50/117 (42.7) | - |
| Cardiovascular disease | | 21/129 (16.3) | - |
| Cerebrovascular disease | | 8/117 (6.8) | - |
| Chronic lung disease | | 4/117 (3.4) | - |
| Asthma | | 7/129 (5.4) | - |
| Active and former smokers | | 22/129 (17.05) | - |
| Chronic renal disease | | 8/117 (6.8) | - |
| Cancer | | 7/129 (5.4) | - |
| Immunosuppression | | 1/117 (0.9) | - |
| **Initial CURB-65** | **median (IQR)** | 1 (0-2) | - |
| 0-1 | N/valid N (%) | 97/142 (68.3) | - |
| 2-5 |  | 45/142 (31.7) | - |
| **Initial qSOFA** | **median (IQR)** | 0 (0-1) | - |
| 0 | N/valid N (%) | 55/99 (55.6) | - |
| 1-5 |  | 44/99 (44.4) | - |
| **Initial ISTH-DIC score** | **median (IQR)** | 0 (0-1) |  |
| 0 | N/valid N (%) | 69/129 (53.5) | - |
| 1-4 |  | 60/129 (46.5) | - |
| **MaximumISTH-DIC score** | **median (IQR)** | 1 (0-2) |  |
| 0 | N/valid N (%) | 38/135 (28.1) | - |
| 1-4 |  | 93/135 (68.9) | - |
| 5 |  | 4/135 (3) | - |
| **Evolution to critically ill disease, N/valid N (%)** | | | |
| ARDS | | 46/142 (32.4) | - |
| Thrombosis | | 7/142 (4.9) | - |
| Bleeding | | 6/142 (4.2) | - |
| Exitus | | 13 (9.2) | - |
| **Medications, N/valid N (%)** | | | |
| Corticosteroids | | 59/142 (41.5) | - |
| Tocilizumab | | 34/142 (23.9) | - |
| Lopinavir-Ritonavir | | 83/129 (64.3) | - |
| Hydroxychloroquine | | 93/129 (72.1) | - |
| Azithromycin | | 84/129 (65.1) | - |
| Anakinra | | 1/129 (0.8) | - |
| LMWH | | 114 (80) | - |

**SupportingTable S1.**Demographic and clinical characteristics of COVID-19 patients and healthy controls.

IQR: Interquertile range; ICU: Intensive care unit; ARDS: Acute respiratory distress syndrome; LMWH: low molecular weight heparin

**SupportingTable S2.**Biochemical parameters and blood cell counts of COVID-19 patients at baseline.

|  | COVID-19  N=142 | NORMAL RANGE | ABNORMAL LEVELS |
| --- | --- | --- | --- |
| **Initial biochemical and coagulation parameters, mean ± SD** | | | N (%) abnormal range |
| LDH, IU/L | 510.5 ±209 | 140 – 280 | 122 (85.9) > 280 |
| CRP, mg/dL | 7.9 ±7.9 | 0 - 1 | 106 (74.6) > 1 |
| Fibrinogen, mg/dL | 511.4 ± 176.9 | 150 - 400 | 99 (69.7) > 400 |
| D-dimer, mg/dL | 1277.5 ± 1953.8 | 0 - 500 | 94 (66.2) > 500 |
| PT (%) | 89.9 ±19.6 | 75 - 100 | 25 (17.6) < 75 |
| aPTT, ratio | 1.1 ± 0.2 | 0.8 – 1.3 | 8 (5.6) > 1.3 |
| **Initial blood cell count, mean ± SD** | | | N (%) abnormal range |
| Platelets/μL | 212953±87817 | 150000 – 450000 | 35 (24.6) < 150000 |
| Neutrophils/μL | 5101± 2801 | 1500 - 8000 | 15 (10.6) > 8000 |
| Lymphocytes/μL | 1052± 621 | 1000 - 4800 | 66 (46.5) < 1000 |

SD: Standard deviation; LDH: Lactate deshydrogenase; IU: International units; CRP: C-reactive protein; PT: Prothrombin time; aPTT: Activated Partial Thromboplastin Time

**SupportingTable S3.**Spearman’s rho correlation of NET and VWF/ADAMTS13 markers in the first available samplewith analytical parameters at baseline.

|  | | cfDNA | citH3-DNA | VWF:Ag | VWF:CB | ADAMTS13 |
| --- | --- | --- | --- | --- | --- | --- |
| PT | Correlation coefficient | -0.288 | -0.159 | -0.345 | -0.189 | 0.362 |
|  | Sig. (two-tailed) | 0.001 | 0.058 | 0.000 | 0.025 | 0.000 |
|  | N | 141 | 142 | 142 | 141 | 142 |
| Fibrinogen | Correlationcoefficient | -0.003 | -0.030 | 0.225 | 0.141 | -0.22 |
|  | Sig. (two-tailed) | 0.975 | 0.731 | 0.009 | 0.108 | 0.816 |
|  | N | 132 | 133 | 133 | 132 | 133 |
| D-dimer | Correlationcoefficient | 0.335 | 0.162 | 0.243 | 0.161 | -0.041 |
|  | Sig. (two-tailed) | 0.000 | 0.059 | 0.004 | 0.051 | 0.636 |
|  | N | 136 | 137 | 137 | 137 | 137 |
| LDH | Correlationcoefficient | 0.435 | 0.134 | 0.325 | 0.247 | -0.63 |
|  | Sig. (two-tailed) | 0.000 | 0.131 | 0.000 | 0.005 | 0.482 |
|  | N | 127 | 128 | 128 | 127 | 128 |
| CRP | Correlationcoefficient | 0.341 | 0.233 | 0.401 | 0.342 | -0.176 |
|  | Sig. (two-tailed) | 0.000 | 0.008 | 0.000 | 0.000 | 0.047 |
|  | N | 127 | 128 | 128 | 127 | 128 |
| PCT | Correlationcoefficient | 0.158 | 0.086 | 0.222 | 0.123 | -0.118 |
|  | Sig. (two-tailed) | 0.087 | 0.354 | 0.015 | 0.185 | 0.202 |
|  | N | 118 | 119 | 119 | 118 | 119 |
| Glucose | Correlationcoefficient | 0.246 | 0.242 | 0.255 | 0.222 | -0.175 |
|  | Sig. (two-tailed) | 0.008 | 0.009 | 0.006 | 0.017 | 0.059 |
|  | N | 116 | 117 | 117 | 116 | 117 |
| Creatinine | Correlationcoefficient | 0.136 | 0.166 | 0.208 | 0.147 | -0.105 |
|  | Sig. (two-tailed) | 0.147 | 0.074 | 0.024 | 0.116 | 0.267 |
|  | N | 116 | 117 | 117 | 116 | 117 |
| Calcium | Correlationcoefficient | -0.139 | 0.068 | -0.343 | -0.131 | 0.094 |
|  | Sig. (two-tailed) | 0.254 | 0.579 | 0.004 | 0.284 | 0.439 |
|  | N | 69 | 70 | 70 | 69 | 70 |
| Platelets | Correlationcoefficient | 0.119 | 0.145 | -0.061 | 0.014 | 0.019 |
|  | Sig. (two-tailed) | 0.158 | 0.085 | 0.474 | 0.873 | 0.823 |
|  | N | 141 | 142 | 142 | 141 | 142 |
| Neutrophils | Correlationcoefficient | 0.241 | 0.233 | 0.341 | 0.246 | -0.082 |
|  | Sig. (two-tailed) | 0.004 | 0.005 | 0.000 | 0.003 | 0.332 |
|  | N | 141 | 142 | 142 | 141 | 142 |
| Lymphocytes | Correlationcoefficient | -0.160 | -0.092 | -0.277 | -0.112 | -0.192 |
|  | Sig. (two-tailed) | 0.074 | 0.304 | 0.002 | 0.212 | 0.030 |
|  | N | 126 | 127 | 127 | 126 | 127 |
| Neutrophil/  Lymphocyte ratio | Correlationcoefficient | 0.265 | 0.179 | 0.409 | 0.208 | -0.156 |
|  | Sig. (two-tailed) | 0.003 | 0.044 | 0.000 | 0.020 | 0.08 |
|  | N | 126 | 127 | 127 | 126 | 127 |

cfDNA: circulating-free DNA; citH3-DNA: citrullinated histone H3 - DNA complexes;VWF: Ag and VWF:CB: antigen levels and collagen binding capacity of von Willebrand factor; AD13: ADAMTS13;PT: Prothrombin time;LDH: Lactate deshydrogenase; CRP: C-reactive protein; PCT:procalcitonin.

SUPPORTING FIGURES


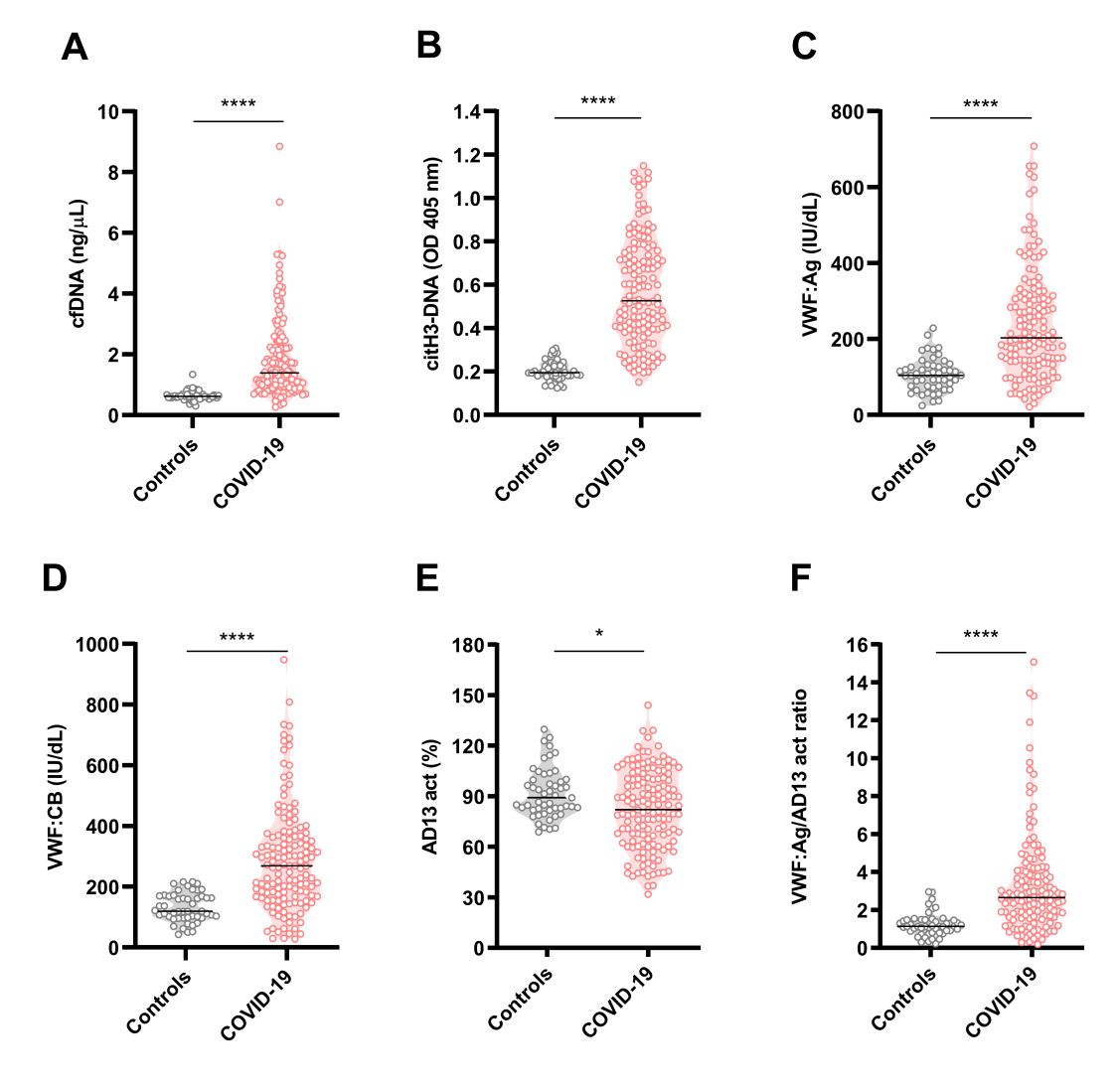


**Figure S1. NETs and VWF/ADAMTS13 in COVID-19 patients and controls.**Plasma from COVID-19 patients (N=142) and from healthy controls (N=50) was obtained and the following markers were measured: **(A)** cfDNA, **(B)** citH3-DNA complexes,**(C)**VWF:Ag, **(D)** VWF:CB,**(E)** ADAMTS13 activity (AD13 act)**, (F)**VWF:Ag/ADAMTS13 activity ratio. Mann-Whitney U-test was used for the statistical analysis. *p<0.05, ****p<0.0001.


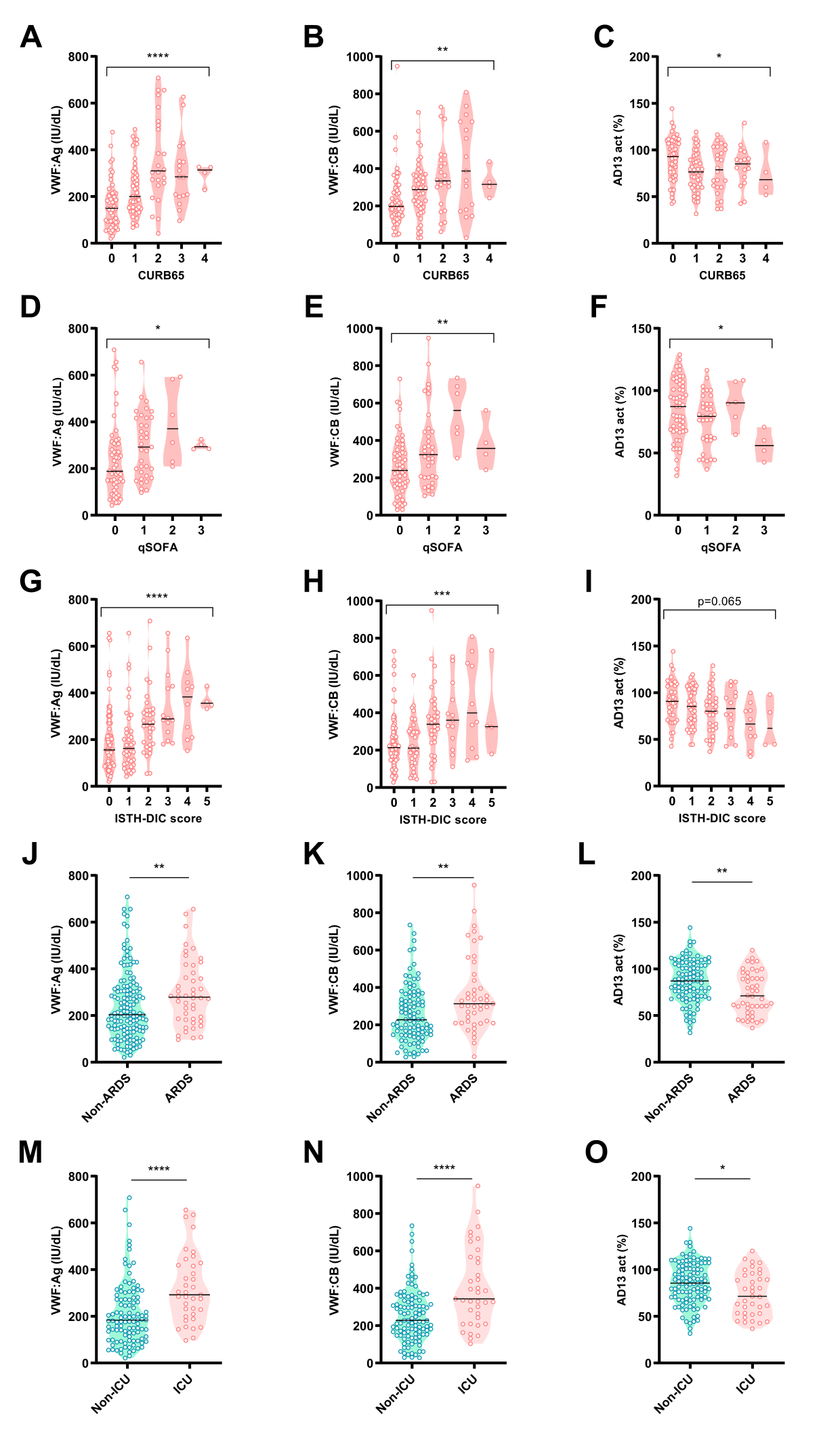


**Figure S2. Severity and coagulopathy scores, ARDS development and need for ICU admission were associated with VWF/ADAMTS13 axis imbalance.** The following markers were measured in the first available plasma sample from COVID-19 patients and analyzed according to CURB-65 score:**(A)**VWF:Ag, **(B)** VWF:CB, **(C)**ADAMTS13 activity(AD13 act);qSOFA score: **(D)**VWF:Ag, **(E)** VWF:CB, **(F)** ADAMTS13 activity; and maximum ISTH-DIC score reached during follow-up:**(G)**VWF:Ag, **(H)** VWF:CB, **(I)** ADAMTS13 activity. Kruskal Wallis test was used for statistical analysis. The same markers were compared in plasma samples from ARDS *vs.* non-ARDS COVID-19 patients: **(J)**VWF:Ag, **(K)** VWF:CB, **(L)**ADAMTS13 activity; and from ICU *vs.* non-ICU patients: **(M)**VWF:Ag, **(N)** VWF:CB, **(O)** ADAMTS13 activity. Mann-Whitney U-test was used for the statistical analysis.*p<0.05, **p<0.01, ***P<0.001, ****p<0.0001.


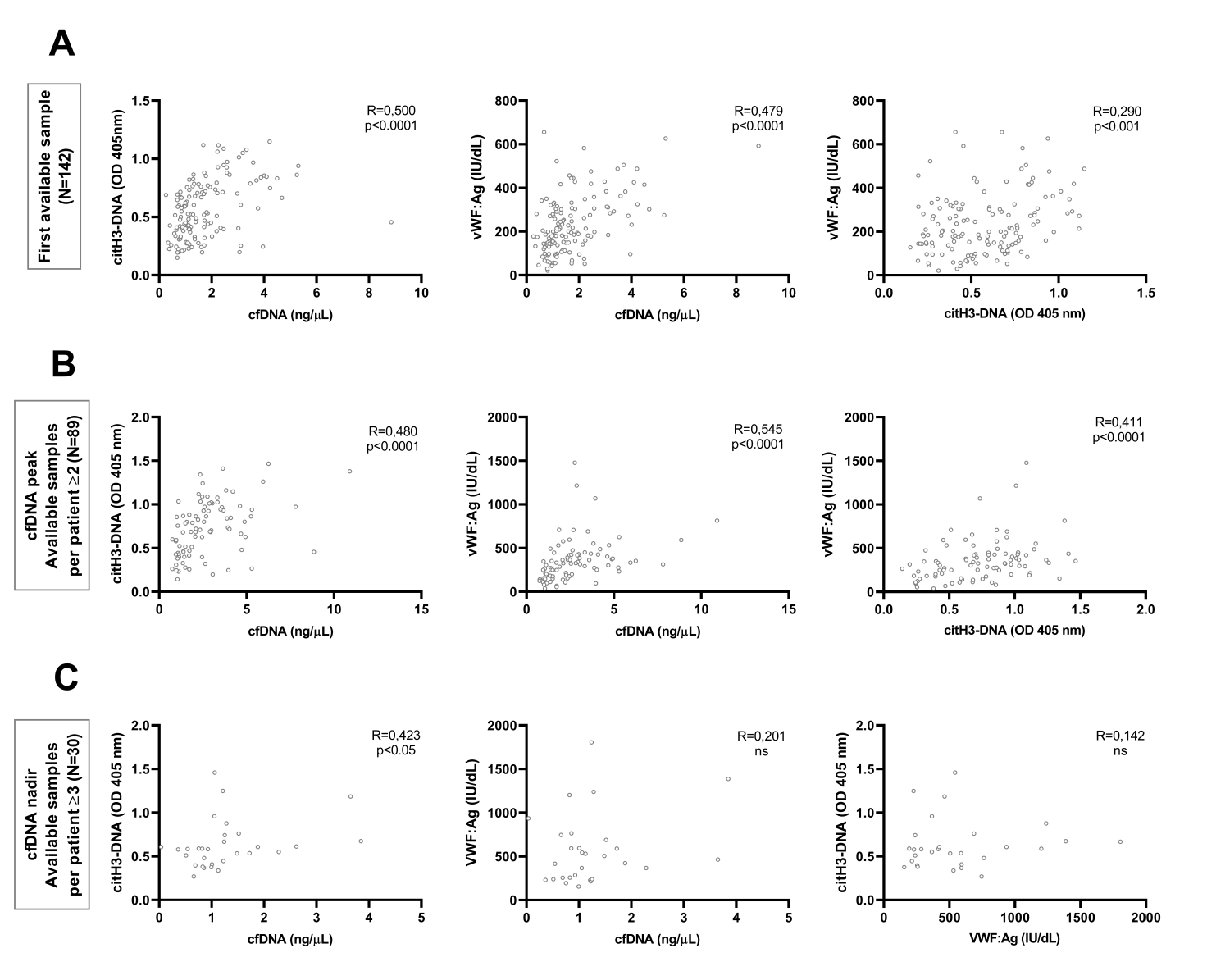


**Figure S3. Correlation between cfDNA, citH3-DNA, and VWF:Ag.** Spearman’s rho correlation test was performed to measure the strength of association between NET markers and VWF at three different time points along COVID-19 progression. **(A)**cfDNA, citH3-DNA, and VWF significantly correlated each other in the firstavailable plasma sample of COVID-19 patients (N=142). **(B)** Higher correlation was observed between cfDNA, citH3DNA, and VWF:Ag in samples exhibiting the maximum cfDNA levels (cfDNA peak) (N=89). (**C)** No correlation was observed between NET markers and VWF:Ag, but cfDNA-citH3-DNA complexes correlation was still maintained in cfDNA nadir (N=30).

**
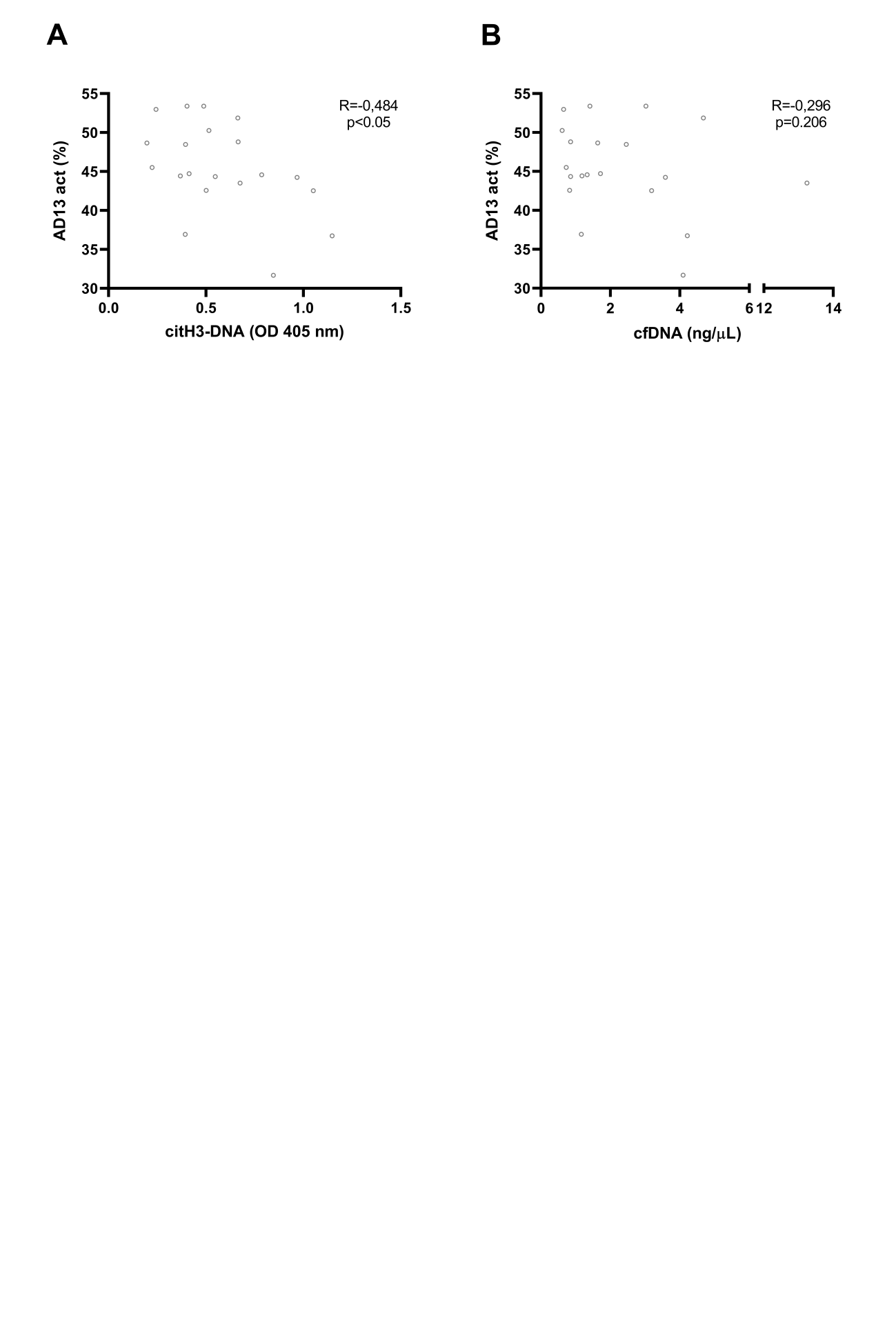
**

**Supporting Figure S4.Correlation between ADAMTS13 activity and NET markers in COVID-19 patients with the lowest ADAMTS13 activity.**Spearman’s Rho correlation test was performed between ADAMTS13 activity and NET markers measured in the first available sample of COVID-19 patients.Inverse correlation between ADAMTS13 activity and citH3-DNA complexes was foundin patients with ADAMTS13 activity lower than 55% (N=16).


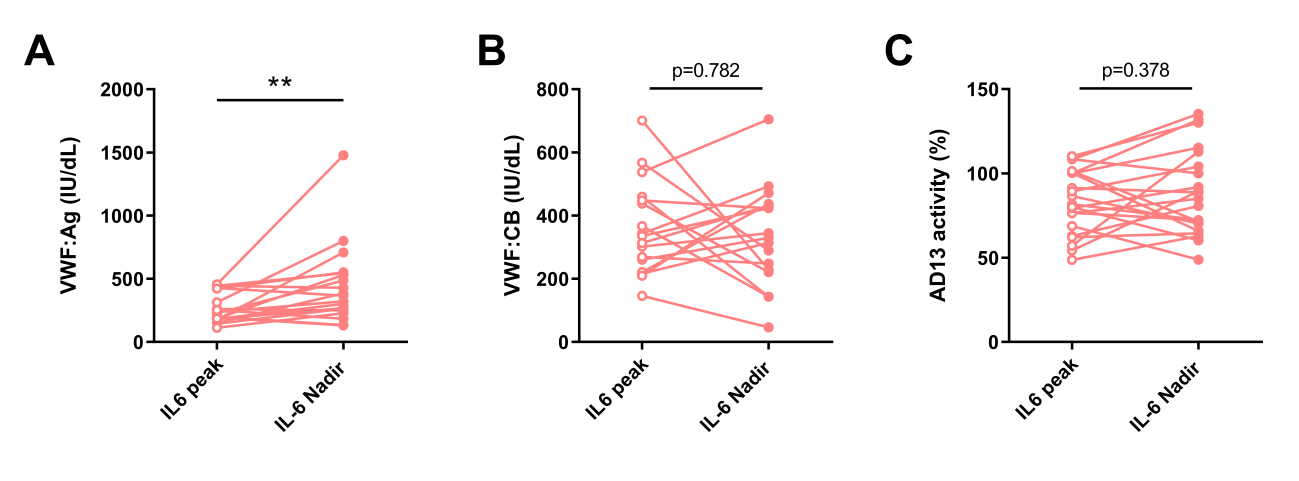


**Figure S5. Evolution of the VWF/ADAMTS13 axis during IL-6 fall.**The following markers were measured in plasma from COVID-19 patients in theIL-6 peak and in the IL-6 nadir (N=19): **(A)** VWF:Ag, **(B)** VWF:CB, **(C)**ADAMTS13 activity (AD13 act). Wilcoxon test was used to analyze the statistical differences between matched paired samples. **p<0.01.
